# Grafting Techniques towards Production of Peptide-Tethered Hydrogels, a Novel Class of Materials with Biomedical Interest

**DOI:** 10.3390/gels1020194

**Published:** 2015-10-21

**Authors:** Mariana Barbosa, M. Cristina L. Martins, Paula Gomes

**Affiliations:** 1 UCIBIO-REQUIMTE, Departamento de Química e Bioquímica, Faculdade de Ciências, Universidade do Porto, P-4169-007 Porto, Portugal; E-Mail: mariana.barbosa@fc.up.pt; 2 i3S, Instituto de Investigação e Inovação em Saúde, Universidade do Porto, P-4200-135 Porto, Portugal; E-Mail: cmartins@ineb.up.pt; 3 INEB-Instituto de Engenharia Biomédica, Universidade do Porto, P-4150-180 Porto, Portugal; 4 Instituto de Ciências Biomédicas Abel Salazar, Universidade do Porto, P-4169-007 Porto, Portugal

**Keywords:** hydrogels, functionalization, peptides, tissue engineering

## Abstract

In recent years, new highly functional polymeric biomaterials are being developed to increase the therapeutic efficacy in tissue regeneration approaches. Peptides regulate most physiological processes and display several other biological activities. Therefore, their importance in the field of biomedical research and drug development is rapidly increasing. However, the use of peptides as therapeutic agents is restricted by some of their physicochemical properties. The development of improved routes of delivery of peptide-based therapeutics is crucial and is crucial and its biomedical value is expected to increase in the near future. The unique properties of hydrogels triggered their spreading as localized drug depots. Several strategies, such as the carbodiimide chemistry, have been used to successfully immobilize bioactive peptide sequences into the hydrogels backbone. Peptide tethering through the so-called “click” chemistry reactions is also a highly promising, yet underexplored, approach to the synthesis of hydrogels with varying dimensions and patterns. The present review focus on the approaches that are being used for the establishment of chemical bonds between peptides and non-peptidic hydrogels throughout the last decade.

## 1. Introduction

In recent years, there has been significant progress in the development of polymers for biomedical applications. New highly functional biomaterials are being designed to increase the therapeutic efficacy in tissue regeneration approaches. In natural tissues, cells are surrounded by a three-dimensional (3D) extracellular matrix (ECM) composed of several biochemical and mechanical signals responsible for modulating their behavior, namely cell attachment, proliferation and differentiation. Fibrous proteins, such as fibronectin, collagen, and laminin, are found in the ECM constitution and are responsible for providing mechanical support. Moreover, ECM acts as a reservoir of cell signaling molecules, such as adhesion molecules and growth factors. Therefore, new generations of biomimetic and bioinstructive materials should act as 3D templates for cell culture, mimicking the ECM environment and promoting cell-matrix interactions responsible for modulating cellular activity and tissue organization [1,2,3].

Understanding the composition and functions of the ECM is of chief importance for developing new 3D cell culture platforms. The incorporation of specific cell signaling molecules, such as growth factors and ECM proteins, into these scaffolds is still a major challenge. These signals are usually adsorbed or covalently attached to a scaffold material, however, prolonged biological activity is thwarted by stability problems after administered into the body [2,3]. An effective functionalization is also dependent on the biomaterial propensity for functionalization and how those modifications will affect its properties. For these reasons, the development of new biomimetic polymers with tunable physicochemical characteristics, according to the desired application, and also capable of being easily functionalized with bioactive building blocks is highly needed. Hydrogels, due to their unique physicochemical properties and unique swelling behavior, are being widely used for tissue engineering applications. Peptides and polypeptides domains have been used to functionalize polymer-based materials in order to obtain new materials with controllable structure, degradability and stimuli sensitive properties. Therefore, this approach is currently being used for the synthesis of highly multifunctional polymeric scaffolds with controllable assembly and characteristics. Peptide-based materials are now attractive candidates for biomedical used due to the progresses observed in synthesis methods and characterization techniques [1,4,5,6].

The aim of this review is to identify the recent approaches used to covalently bound peptides to hydrogels, describing advantages and limitations of each strategy [7], with particular emphasis on “click” chemistry techniques. These approaches are reviewed herein and refer to the last decade, *i.e.*, reports from 2005 to the present date.

## 2. Peptides Underlying a Paradigm Shift in Traditional Therapies

The use of peptides, comprising the functional subunits of proteins, as drug candidates has been fostered over the last decades [8,9]. Peptides regulate most physiological processes, regulating cellular function and coordinating intercellular communication. In fact, specificity of molecular recognition and consequent ligand-binding interactions are determined by specific amino acid sequences of peptides and proteins [10,11]. Moreover, they may have several biological activities such as antimicrobial, antithrombotic, opioid, antioxidant, among others. Consequently, peptides are nowadays an important issue in biomedical research and drug development in various therapeutic classes, ranging from thrombolytics, immunomodulators and growth factors to antimicrobials [8,12,13,14,15,16,17]. Peptides have significant advantages over other small molecules in terms of specificity/affinity for targets and toxicity profiles, and over antibodies in terms of tissue penetration and immunogenicity owing to their smaller size [18,19,20,21]. Moreover, peptides are generally biocompatible and do not cause severe immune responses, particularly those with smaller sequences, as they are composed of naturally occurring or metabolically degradable amino acids. In general, the compositional homology between peptide drug candidates and its bioactive parent molecules significantly diminishes the risk of unpredicted side-reactions and the production complexity, thus lowering the production costs [11,22]. Furthermore, peptides are also very amenable to site-specific modifications that might be used to tailored specific properties [21].

Consequently, a large number of peptide-based drugs are now being marketed and the number of candidates entering clinical evaluation in recent years is steadily increasing [12,22]. Bioactive peptides and peptidomimetics compose several marketed drugs used against most diverse diseases, such as, just to name a few: the anti-HIV-1 agent Enfuvirtide, the natridiuretic peptide Nesiritide (used to treat acute congestive heart failure), antimicrobial peptides such as gramicidin D (component of the topical antibiotic drug line Neosporin^®^), peptide hormones like Oxytocin (labor induction agent) or Leuprolide (gonadotropin-releasing hormone analogue), or even the bone density conservation agent salmotonin (salmon calcitonin), which is the active ingredient of antiosteporotic drugs like Miacalcin^®^ from Novartis [8,22]. Therefore, the synthesis of such structures has been a major focus of organic chemistry for over a century in order to improve the prospects for synthetic therapeutic peptides [18,23].

The use of peptide therapeutics is expected to increase in the near future. More will go into clinical trials, some will be produced with increased potency and/or specificity, and new conjugated forms (for example with polysaccharides or synthetic polymers) will be designed expanding the range of targets [22,24]. Moreover, peptides are expected to find increased biomedical applications not only as the active ingredient of drugs, but also as “add-ons” to other therapeutic compounds or biomaterials. In this context, peptides can be used as targeting moieties, as carriers to provide transport across cellular membranes, and to modify the bioactivity of the original compound/material. In the field of biomaterials, peptides have been extensively used as cell-instructive motifs with different roles, namely, to promote cell-adhesion to otherwise non-adhesive polymers [25,26]. Besides its active role in ligand–receptor interactions, peptides can also promote protein–protein interactions and antibody detection [27]. One of the most interesting applications is the development of drug delivery carriers, since peptides can be used as stimuli-sensitive linkers that can be used for controlled drug release in the presence of certain enzymes, which allows the delivery of pharmaceuticals in very specific locations and conditions. In this context, enzyme-sensitive hybrid materials composed of synthetic or natural polymers and peptide/protein domains, which respond to specific proteases, have been prepared using genetic engineering and/or chemical approaches [26,28].

Finally, peptides alone are been successfully used as innovative biomaterials. One important example is the novel group of materials named self-assembling peptides (SAPs), which self-assemble into hydrogen-like nanostructures. Stupp’s group has developed peptide amphiphiles (PA) by combining a hydrophobic block, usually an alkyl chain, a β-sheet forming peptide responsible for the self-assembling and a third section with the bioactivity molecules, such as peptides. These PA are capable of self-assemble and form highly ordered gels under physiological concentrations of salt solutions [29,30,31,32]. For instance, the RADA16-I (AcN-RADARADARADARADA-CONH_2_) peptide can undergo spontaneous assembly into an organized network of nanofibers under physiological conditions, forming an ECM-like hydrogel for the encapsulation and delivery of growth factors and cells [33,34]. Consequently, these peptide-based hydrogels have been finding numerous applications in the biomedical field due to its functional supramolecular structure capable of forming 3D matrices [25,29,30,35].

## 3. Peptide Delivery Systems

The use of peptides as therapeutic agents is restricted by some of their physicochemical properties. The large molecular weight of peptides influences their diffusion through the epithelial layer, which leads to low bioavailability. Moreover, peptides are mostly hydrophilic so the transfer across biological membranes by passive diffusion is limited. Peptides can also undergo aggregation, adsorption and denaturation and are also vulnerable to proteolytic cleavage so their stability in the blood stream and concentration *in vivo* is limited [36,37]. Hence, progression of peptide-based compounds into clinical therapy is thwarted by stability problems and short circulating plasma half-life [11,38]. In fact, peptides are based on amino acid building blocks and thus can be rapidly inactivated or eliminated after administered into the body. Even when administered parenterally, they can be rapidly metabolized by peptidases or cleared from circulation by the kidney, spleen, or liver [37,39].

The development of improved routes of delivery for peptide-based therapeutics is crucial and it biomedical value is expected to increase in the near future [11,38]. Research is focusing on improved routes of delivery that are expected to open up the potential of peptide drugs. In order to overcome the aforementioned drawbacks and extend the bioactivity of therapeutic peptides *in vivo*, it is possible to use a delivery matrix that protects peptides from neutralization and degradation*.* Cell-signaling peptides can act as tethered ligands and be cross-linked with matrix scaffolds by a plethora of chemical bonding strategies. The matrix can provide controlled peptide delivery at a specific site or systemically in a continuous manner, preventing repeated administrations of the drug. The use of tunable peptide delivery system is of major importance to achieve controllable dosage for higher effectiveness or to provide a sustained release during the course of the treatment [38,40]. Finally, by limiting the delivery to specific target sites and avoiding healthy tissues and cells the efficacy of the drug is improved and eventual toxic effects at non-target sites can be prevented [41,42,43].

Polymers are an ideal class of materials to prepare drug delivery systems, since they are quite versatile and their physicochemical properties, such as biocompatibility, biodegradability, network structure and mechanical strength, are easily adapted and tuned for a particular application. It is possible to customize the material by, for example, altering its molecular components, adjusting the polymerization conditions, or modifying the original polymer with new bioactive compounds [40,41,44]. When developing a drug-releasing polymer scaffold, several criteria should be addressed, namely the drug release profiles, the drug-loading capacity and binding affinity of the polymer and the spatial distribution of the bioactive compound within the matrix backbone. It is also important to consider how the incorporation of the drug into the polymer will affect its bioactive properties [45].

## 4. Hydrogels as Drug-Delivery Vehicles and Scaffolds for Tissue Regeneration

Current tissue engineering strategies comprises both cells and a matrix-scaffold. Therefore, it is essential to select a suitable biomaterial taking into account the envisioned application and the characteristics of the tissue (e.g., stiffness, chemical composition, biological signals) [29]. The aforementioned scaffolds can be fabricated from either biological materials or from synthetic polymers. Biological scaffolds interact with resident cells providing biofunctional cues that modulate cellular behavior. However, they are structurally complex and present a high variability of cell-signaling cues making it difficult to precisely control cellular activity. On the other hand, synthetic scaffolds are usually not bioactive, providing inadequate biological information for cell culture. Nevertheless, they found many applications in the field of tissue engineering because they allow for precise control of their mechanical properties. In this regard, the ideal scaffold for biomedical applications should be developed combining the physicochemical properties of both synthetic and natural polymers [46,47].

Hydrogels have been broadly investigated as biomaterials for tissue engineering strategies, in which they are used as scaffolds, drug delivery systems, as well as 3D cell culture platforms [3,48,49]. Hydrogels are hydrophilic polymeric networks with 3D configuration that can retain a significant amount of water or biological fluids. Hydrogels generally possess excellent biocompatibility due to the tissue-like physicochemical properties and their ability to swell under biological conditions [48,50,51,52,53,54]. Hydrogels recreate the hydrated microenvironments and the structure of the ECM where cells are embedded in real 3D conditions, therefore they have been widely used as scaffolds [55]. When used as artificial ECM, hydrogels may act as a substitute of natural tissues by rearranging cells into an ordered scaffold to support the newly-forming tissues, and a hydrated space for diffusion of nutrients, oxygen and metabolites [56,57,58].

The presence of hydrophilic groups such as –OH, –CONH–, –CONH_2_, –SO_3_H in polymer chains is responsible for their ability to absorb water. The water content depends on the nature of the aqueous environment and polymer composition, and is the key factor which determines the physicochemical characteristics of the hydrogel [51,59,60,61]. The elastic nature of completely swollen hydrogels has been found to diminish the risk of irritation to the adjacent tissues after implantation. Non-specific protein adsorption and cellular adhesion, followed by increased risk of an immunological reaction, are prevented by the low interfacial tension between the hydrogel surface and the surrounding biological components [62]. Hydrogels are also used for cell encapsulation due to their high permeability which allows diffusion of nutrients, oxygen and cell metabolites [49,52,63].

Hydrogels are usually prepared under mild reaction conditions without need for organic solvents, at ambient temperatures [51]. Cells can be uniformly seeded within the interstitial pores created in the hydrogel network [49,64]. The density of those pores can be adjusted during the polymerization reactions, namely the affinity of hydrogels for the swelling solvent and the cross-linking density within the matrix. In addition, it is possible to load bioactive drugs and biomolecules into the gel matrix, protecting them from degradation, and subsequently release them at a diffusion-dependent rate. Actually, controlling the hydrogel structure is the key factor to customize the release schedule allowing them to be either used for systemic delivery or to preserve the appropriated bioactive concentration around target site [49,53,65].

Hydrogels are extremely stable in the presence of high amounts of water, however, when desired, they can be designed to be sensitive to external stimulus such as the presence of enzymes or certain environmental conditions (e.g., pH, temperature, or electric field). The physical structure and bio-adhesive properties of hydrogels allows them to adapt and adhere to the surface to which they are applied, and depending on their bio-adhesive properties, they can be immobilized in [53,66].

However, hydrogels are also associated to several limitations. Natural-derived hydrogels are usually associated with low tensile forces which can promote its degradation or moving away from the desired application site, making them inappropriate for load-bearing applications. This restriction may not be critical in the traditional parental drug administration. Problems related to drug delivery properties of hydrogels are still a major concern. The drug-loading capacity and distribution of the bioactive compound, especially non-soluble drugs, within the hydrogel network may be limited. Promoting a faster drug delivery rate can be achieved by increasing pore sizes and water content of the hydrogel. Most hydrogels present deformable features and can be easily administrated by injection, otherwise they require surgical implantation. These limitations can restrict the application of hydrogels in the development of drug delivery systems [53].

There are different types of hydrogel-forming polymers generally divided into two categories according to their source, natural or synthetic, each presenting advantages and limitations. Natural hydrogels have been widely used for tissue engineering approaches and are synthesized from proteins and ECM components like collagen, fibrin, hyaluronic acid or Matrigel, or from biological sources such as agarose, alginate, chitosan, silk fibrils. Natural polysaccharides display endogenous factors responsible for modulating several cellular functions, such as adhesion, viability and proliferation. Furthermore, being biodegradable, they can be replaced by *bona fide* ECM over time. Therefore, natural hydrogels can act as scaffolds for cellular guidance and wound healing [3,5,48,63,67].

Still, natural polymeric hydrogels are complex and exhibit a plethora of cell-signaling molecules and exhibit large batch to batch variability, making it difficult to correctly define the cell-modulating signals, to tune the material physicochemical properties and to attain highly reproducible results using such scaffolds. On the other hand, synthetic polymers, such as poly(ethylene glycol) (PEG), poly(acrylic acid) (PAA), poly(2-hydroxyethyl methacrylate) (PHEMA), poly(vinyl alcohol), (PVA) and polyacrylamide (PAAm), have emerged as an important alternative due to its reproducible properties and controllable physical properties. Synthetic hydrogels can act as a blank (*i.e.*, without cell-binding ligands) scaffold for cell culture as they maintain the viability of encapsulated cells and allow ECM deposition as they degrade. However, most synthetic hydrogels alone lack bioactivity and cell signaling motifs and only function as passive scaffolds for cells [3,63,68].

Limitations of both natural and synthetic hydrogels have motivated the development of new synthetic approaches and crosslinking strategies to modify these polymers with the essential biophysical and biochemical signals to match the physiological cellular environment [3,5,48,49,51,67]. In addition, these materials may be decorated with biochemical signals that bind to specific cell receptors and modulate cell behavior [57,69]. *In situ*-forming hydrogels present the added benefit of injectability, and can be used to fill tissue defects with irregular patterns in a minimally invasive manner [58]. Overall, the unique properties of hydrogels triggered their spreading as localized drug depots. They form highly hydrated 3D networks, with a selective permeability that affords some control over drug release rates, which in addition may be triggered intelligently by interactions with biomolecular stimuli. Hydrogels are typically biocompatible since they possess native tissue-like properties [41,55]. Moreover, hydrophilic biomolecules, namely peptides, are compatible with hydrogels [56,70,71].

Polymers can be physical or chemical cross-linked to originate hydrogel-based scaffolds with distinct composition, networks and water solubility. Those characteristics affect other relevant properties such as the swelling degree and the degradation behavior. By controlling the physicochemical properties of hydrogels, the delivery kinetics of a drug can be adjusted to the desired rate. They can be prepared from soluble precursor solutions that crosslink *in situ* under mild conditions. Specific bioactive agents can be loaded into hydrogels, using different strategies involving physical or chemical interactions. Drug entrapment can be achieved either through drug trapping during hydrogel formation, or drug absorption by pre-fabricated hydrogels. If the drug of interest is added to the polymer solution before crosslinking, it becomes entrapped within the network, generally retaining full bioactivity [41]. However, when a drug is physically loaded into a hydrogel matrix, assure a long-term continuous delivery is difficult since the drug is essentially released by diffusion. Therefore, in order to achieve a sustained drug release it is necessary to improve the chemical interactions of hydrogels and bioactive compounds. Another aspect to consider, is the degradation rate of hydrogels which greatly influence their drug delivery behavior [41,57]. Moreover, drug-containing biomaterials can also be programmed to release the drug at a specific site in response to a particular biological milieu [72].

The emergence of stimuli-sensitive hydrogels as gained special interest in the field of tissue regeneration and biomedical engineering due to their ability to undergo structural modifications and act as controllable drug-release systems in response to environmental changes. Also known as smart hydrogels, they are developed to recognize both physical (temperature, light, mechanical tension) and chemical (pH, biomolecules, biochemical environment) stimulus [41]. For local release and higher therapeutic efficacy in tissue-regeneration approaches, the hydrogel carriers may simultaneously act as a tissue-engineering scaffold, as is the case of delivery systems for pro-regeneration drugs, like growth factors. They consist in large polypeptides that bind to specific cell-surface ligands and modulate cellular activity and gene expression [37,73].

Stimuli-responsive polymers have been attractive materials for the drug delivery field. These polymers have the ability to change its properties according to the surrounding environment. As said previously, both physical and chemical stimuli can induce responses in these “smart” systems [26,74]. Jeong *et al*. developed an injectable drug delivery system from an enzymatically degradable polypeptide block copolymer capable of undergo sol-gel transition as the temperature increases [75,76]. These peptide-based biomaterials are not within the scope of this review, however, the reader is referred to a variety of research and review papers that describe the fundamental aspects and application areas of peptide carriers with stimuli-sensitive properties [66,74,77,78,79,80,81]. The covalent coupling of a drug to a polymer, although generally irreversible in nature, may be used for delivery purposes if the carrier is biodegradable or if a labile drug–polymer linker is used. However, the first strategy is often inadequate since the degradation rate in the human body is usually slow and unpredictable. The second approach generally provides a higher degree of versatility and efficacy, as very selective triggering mechanisms can be chosen to enable drug release upon response to specific stimuli. The released drug acts locally to modulate the response of cells, within and/or near the material, activating pro-regenerative functions [73]. If peptide-grafted polymers are subsequently used to prepare hydrogels the rate of peptide release will depend on the cleavage kinetics of the peptide-network linkage, and the rate of peptide diffusion from the matrix, once free from the polymer backbone [82]. At the same time, the hydrogel scaffold acts as an artificial 3D matrix that mimic the natural ECM, promoting an efficient exchange of nutrients, oxygen and cellular metabolites thus providing an adequate cellular microenvironment inducing the repair of injured tissues, and the restoration of natural functions *in situ* [58,83,84].

The immobilization of bioactive peptides onto the backbone of hydrogels derived from synthetic polymers improves cell–matrix interactions. Current approaches allow the use of synthetic PEG hydrogels as scaffolds for cell culture due to its hydrophilic nature and ability to incorporate adhesion-peptides which promotes cell–polymer interactions. For example, functionalized PEG hydrogels with cell-adhesion peptides offers biological matrix functionality and allows cells to interact with the scaffold. The amount of peptide loaded onto the hydrogel and distribution throughout the hydrogel network greatly influences cell adhesion and dispersion. Studies demonstrated that incorporation of peptide-based binding motifs on PEG-based hydrogels promoted binding and proliferation of osteoblasts, fibroblasts and smooth muscle cells. The hydrogel matrix provided an artificial ECM environment to the cells since the incorporated adhesion sequences recognize specific ligands displayed on the cell-surface [85,86]. The tripeptide RGD, found initially in fibronectin, is considered the minimal integrin-binding sequence. This adhesion motif was later identified within collagen, vitronectin, laminin, fibrinogen, among others ECM proteins. Peptide sequences derived from laminin, including GFOGER, IKLLI, LRE, IKVAV, YIGSR, DGEA, and PDSGR, have also been demonstrated to enable cell adhesion, proliferation, and differentiation [85,87]. Reactive acryloyl-PEG-*N*-hydroxysuccinimide was conjugated with RGD peptide by reaction of its amine terminus with succinimide group. The resulting macromere reacted with an *in situ* photocrosslinkable chitosan by free radical photoinitiated polymerization after UV irradiation [86,88].

The use of a combinatorial library of different cell-binding and other matrix analogue peptides is mandatory to induce cellular activity and cell–matrix interactions, in order to promote tissue regeneration [89,90]. Such is the case of peptide domains sensitive to the action of proteases which were loaded into both synthetic hydrogels, such as PEG-polymer chains [91], and natural hydrogels, like alginate [92]. Their cleavage allows to expand the interstitial space of the hydrogel network to promote cell growth and migration, and ECM deposition [86]. For example, covalently immobilized growth factors, like the basic fibroblast growth factor (bFGF), to PEG hydrogels promoted cellular functions involved in the process of tissue formation, namely cellular migration and proliferation [89].

The synthesizing novel cell-culture scaffolds with ECM-like properties in crucial for developing, *in vitro*, environments capable of promoting cellular activity [93,94]. There are several peptide motifs that exhibit biological activity, like cell-adhesion and proteolyzable peptides, or influence mechanical properties, such as elastin-like peptides. Using these bioactive cues allow the precise tuning of the material physicochemical characteristics. Therefore, a plethora of new bioactive peptides, for example structural and cell-signaling sequences, are been studied to improve the field of peptide-based materials [93].

## 5. Peptide Tethering onto Hydrogels through “Click” Chemistry

**Figure 1:**
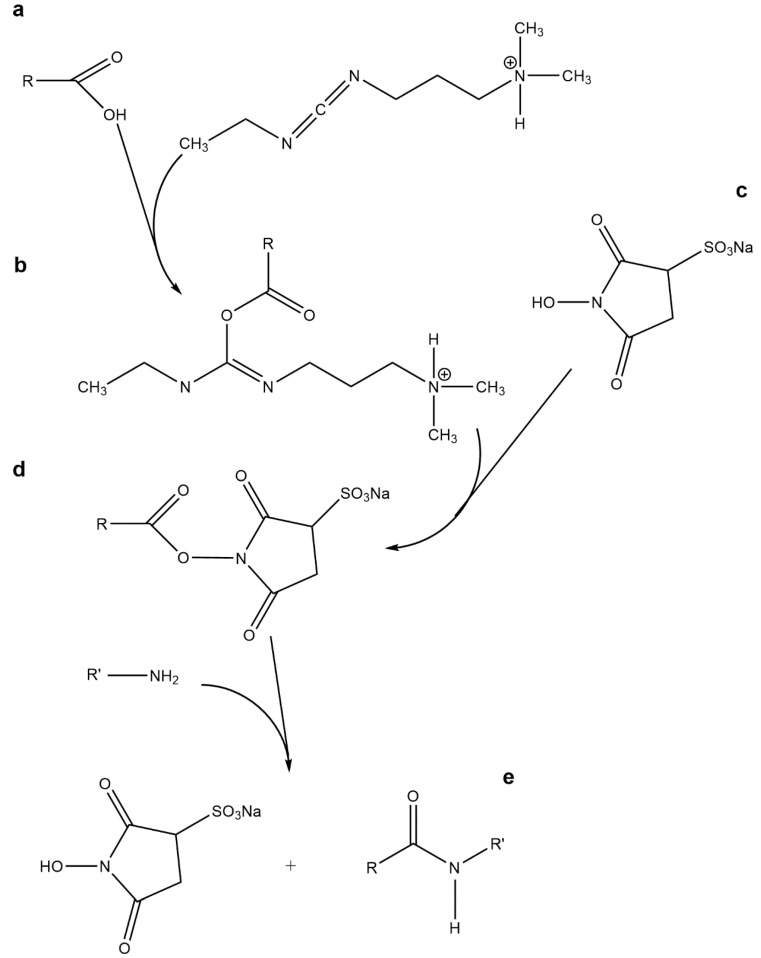
1-Ethyl-3-(3-dimethylaminopropyl) carbodiimide (EDC)-mediated amide formation in the presence of sulfo-*N*-hydroxysuccinimide (NHS): upon carboxyl activation with (**a**) EDC, the resultant intermediate (**b**) *O*-acylisourea reacts with the auxiliary nucleophile (**c**) sulfo-NHS leading to an (**d**) ester intermediate that ultimately reacts with the amine group, yielding the desired (**e**) amide bond.

When small molecular-weight drugs, such as oligopeptides, are loaded into alginate hydrogels simply by physical entrapment, the diffusion-controlled release kinetics is generally too fast. If a more sustained release is to be attained, it might be necessary to conjugate both components via stronger interactions such as covalent bonds [57,95]. In this connection, the water-soluble 1-ethyl-3-(3-dimethylaminopropyl) carbodiimide (EDC), is the carbodiimide of choice for the covalent attachment of proteins and peptides to hydrogels such as alginate, by forming amide linkages between the amine containing biomolecules and the polymer’s carboxylic groups [96]. In this crosslinking reaction, typically, performed between pH 4.5 and 7.5, EDC catalyzes the formation of amide bonds, usually in the presence of an auxiliary nucleophile such as *N*-hydroxysuccinimide (NHS) or *N*-hydroxysulfosuccinimide (sulfo-NHS). When used, the auxiliary nucleophile reacts with the intermediate *O*-acylisourea formed upon carboxyl activation with EDC, leading to a more stable, but still reactive, ester intermediate that ultimately reacts with the amine group. Consequently, coupling reactions mediated by EDC/[sulfo-]NHS (Figure 1) are more effective and high-yielding than with the use of EDC by itself [97,98].

Despite carbodiimide chemistry has been used to successfully immobilize bioactive peptide sequences in alginate [92,99], PEG-heparin hydrogels [100,101] and hyaluronic acid [102], peptide tethering through the so-called “click” chemistry reactions [103] is a highly promising, yet underexplored, approach to the synthesis of hydrogels with varying dimensions and patterns. Sharpless and co-workers formulated in 2001 the concept of “click” chemistry [104] and defined it as a group of highly chemoselective reactions where two functional groups exclusively react with each other, even in the presence of other reactive functionalities, with minimal byproducts. Such reactions are thermodynamically favored (driving force superior to 20 kcal·mol^−1^), and are quite appealing for *in vivo* applications where a diverse range of functionalities is present in aqueous media. Hence, “click” chemistry has been used as a high yield tool towards the immobilization, through covalent interactions, of peptides, bioactive drugs, or fluorescent markers onto biopolymers, following simple reaction routes under mild chemical conditions [105,106,107,108,109,110]. Remarkably, a selection of “click” reactions has been shown to occur efficiently in complex biological media and in the presence of living cells due to their high chemoselectivity. Currently, the fields of application of this type of chemistry are diverse and range from materials engineering and bioconjugation to pharmaceutical sciences and medical imaging and the number is expected to raise in the future [105,106,107,108,109,110].

One of the most studied reaction that fulfills all the criteria for “click” chemistry is the copper(I)-catalyzed azide–alkyne cycloaddition (CuAAC) to produce a stable 1,2,3-triazole linkage between the two “clicked” building blocks. However, during the years several equally effective metal-free strategies have emerged, such as copperless azide–alkyne cycloadditons and Diels–Alder reactions, just to name a few [110,111]. These “click” chemistry approaches are next revised in more detail.

### 5.1. Copper-Catalyzed Azide–Alkyne Cycloaddition (CuAAC)

**Figure 2:**
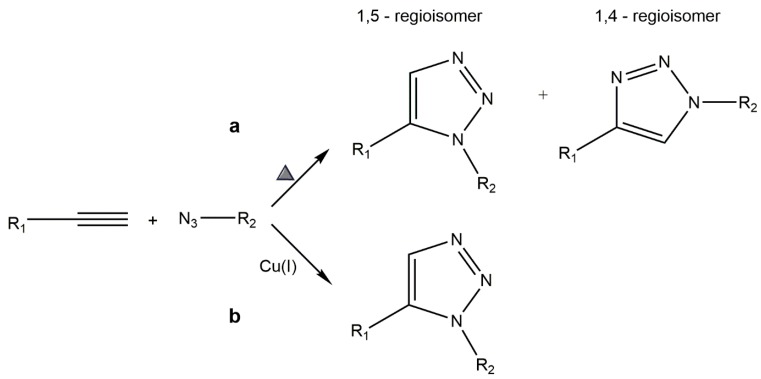
Huisgen’s 1,3-dipolar cycloaddition of azides and alkynes to give triazoles: (**a**) unactivated azide–alkyne cycloaddition yielding a mixture of the 1,4- and 1,5-triazole regioisomers; (**b**) CuAAC leading to regioselective formation of the 1,4-triazole isomer.

The Huisgen’s 1,3-dipolar cycloaddition between azides and alkynes yielding triazoles is gaining interest as an appealing chemoselective approach amongst the “click” reactions family (Figure 2). This reaction is widely used since several molecules can be easily functionalized with both alkyne and azide components which react selectively with each other [112,113,114,115].

The catalyst-free azide–alkyne cycloaddition (Figure 2a), pioneered by Huisgen in 1963, required high temperatures and pressures, since azides and alkynes have low reactivity, at atmospheric pressure and room temperature, towards each other and other functional groups present in the biological milieu. Consequently, this reaction is known to be extremely slow and inactive *in vivo*, due to the aqueous mild conditions. Furthermore, this cycloaddition has low regioselectivity since it gives two different regioisomers, namely the 1,4- and 1,5-triazole, which are extremely difficult to separate. These issues were later overcome by Tornøe and Meldal, who introduced Cu(I) catalysis (Figure 2b) in alkyne-azide coupling reactions, rendering a faster and selective reaction; addition of copper as a catalyst favors formation of only the 1,4-regioisomer [105,107,112,113,114].

The CuAAC reaction, *i.e.*, the copper-catalyzed cycloaddition reaction between alkynes and azides yielding triazoles, occurs effectively under an extensive range of environments and with many Cu(I) sources. Usually, copper(II) salts are used, such as copper sulfate pentahydrate or copper acetate, in combination with metallic copper or sodium ascorbate which act as reducing agents of copper(II) to copper(I) [114,116].

This reaction has attracted much attention for the synthesis and post-polymerization modification of polymers. Both unprotected reactive groups are stable to the synthesis conditions used in the course of solid phase peptide synthesis (SPPS), so they can be easily introduced into the peptide sequences. Several studies confirmed this statement by successfully grafting and immobilizing biomolecules to polymers and synthesizing copolymers [115,117]. The triazole link created between the two building blocks that are coupled is very stable and chemically inert to most reactive conditions. In contrast to amides, and due to their high aromatic stabilization, triazoles are extremely stable to hydrolysis, and are resistant to the activity of both reducing and oxidizing agents, diverging from other aromatic heterocycles, and to metabolic degradation [113,114]. The dipole moment (around 5D) of these heterocycles favors the formation of hydrogen bonds as well as helps them to participate in π stacking and dipole–dipole interactions [112]. Interestingly, triazoles have been found to display diverse biological activities, including anti-HIV and antibacterial activity [118].

It stems from the above that CuAAC are extremely relevant for tissue engineering applications, given not only the simple experimental conditions but also the chemoselectivity, since both reactive groups only react with each other even in the presence of additional functional groups. Such cycloadditions are effective strategies to develop hydrogels for cell-culture due to its mild aqueous reaction conditions, and also as drug release materials since it is relatively easy to load bioactive drugs and other biomacromolecules within the hydrogel network throughout its formation [119]. In addition, CuAAC can also be used in the crosslinking of PEG hydrogels with peptide sequences susceptible to degradation [91]. A chitosan derivative bearing an alkyne moiety was successfully modified with a PEG-like azide through this “click” reaction, proving the facile chitosan conjugation with drugs and other bioactive molecules, such as peptides [120]. The CuAAC reaction was also used to covalently attach a PEGylated peptide with poly(lactide-*co*-ethylene oxide fumarate) (PLEOF) hydrogel [121].

Overall, CuAAC is an extremely valuable tool towards peptide tethering onto hydrogels and other biomaterials. Still, use of the copper catalyst can be problematic in some cases and, especially, towards *in vivo* applications, which underlies recent interest in copper-free azide–alkyne click reactions [91].

### 5.2. Strain-Promoted Azide–Alkyne Cycloaddition (SPAAC)

CuAAC has been used successfully in the *in vitro* modification of biomacromolecules and also in the labeling of bacterial and mammalian cells, however, the negative effects associated with the required copper catalyst is a major limitation for its *in vivo* application. This has promoted not only the optimization of CuAAC bioconjugation strategies suitable for *in vivo* applications, but also the development of azide–alkyne cycloaddition protocols, without the need for copper or other cytotoxic catalysts [122,123].

**Figure 3:**
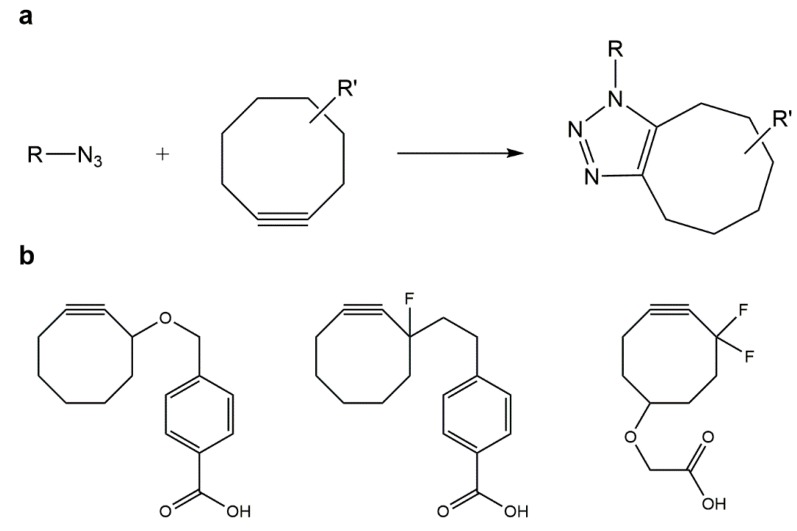
(**a**) Strain-promoted azide–alkyne cycloaddition (SPAAC); (**b**) substituted cyclooctynes currently employed to lower the activation barrier of azide–alkyne cycloadditions, thus avoiding use of copper catalysts.

The Bertozzi group developed a strain-promoted azide–alkyne cycloaddition (SPAAC) reaction (Figure 3a) for the bioorthogonal chemoselective modification of biomolecules and living cells. As proven by Bertozzi and colleagues, cyclooctynes ring strain is responsible for lowering the aforementioned activation barrier of azide–alkyne cycloadditons, surpassing the need of the copper catalyst. Furthermore, they performed successful cycloaddition reactions between several low molecular weight compounds and novel substituted cyclooctynes (Figure 3b). SPAAC is characterized by its simplicity and great orthogonality which promoted the spread of this approach from biomedical and polymers science to materials engineering [122,123,124].

In the past years, the Cu-free reaction between several cyclooctyne derivatives and azides was found to be extremely fast due to the previously mentioned ring strain and also to electron-withdrawing effects from fluorine substituents. However, the synthesis of cyclooctyne is comprised of over ten steps with a low overall yield, which makes this strategy unsuitable for large-scale synthesis [108]. Moreover, insertion of cyclooctyne-based building blocks in peptides is incompatible with current procedures in solid-phase peptide synthesis (SPPS), since cyclooctynes are highly reactive, especially with the acidic compounds used in the final cleavage/deprotection steps in SPPS. This may be circumvented by alternative synthesis of azido-peptides to be subsequently reacted with cyclooctyne-modified scaffolds; in this connection, DeForest and Anseth developed a SPAAC “click” reaction between a terminal difluorinated cyclooctyne (DIFO)-PEG hydrogel and a bis(azide) di-functionalized polypeptide [125].

### 5.3. Thiol-ene “Click” Chemistry

The thiol-ene chemistry occurs between thiols and carbon–carbon double bonds, also known as “enes”. This highly reactive reaction involves either a radical mediated addition or an anionic chain process, the so-called thiol Michael addition [126].

**Figure 4:**
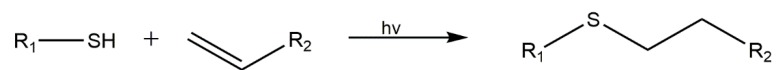
Radical-mediated thiol-ene chemistry: the tiol-ene “click” reaction involves the addition of a thiol to a double bond under light irradiation (h*ν*).

Radical mediated thiol-ene chemistry occurs under light irradiation (Figure 4) towards incorporation of any biomolecule containing a thiol group and is efficient, high yielding and highly flexible [108,126,127,128]. In addition, it allows to obtain a homogeneous network through a step-growth mechanism controllable by standard lithographic processes. The cytocompatible polymerization conditions used make this technique suitable to develop 3D culture platforms. A multi-armed thiolated PEG was modified with alkene- and acrylate-functionalized small molecules via UV-initiated thiol-ene coupling chemistry. This technique was used to form peptide-functionalized PEG hydrogels [127,128,129]. The incorporation of an enzyme-sensitive linker into a norbornene-functionalized PEG rendered hydrogels with controllable rates of degradation [91] and with both enzymatically degradable peptide and adhesive peptide, CRGDS, originated cell- and enzyme-responsive hydrogels [127,130]. Other example is the introduction of biochemical cues by thiol-ene photoconjugation in a PEG-based hydrogel previously formed by SPAAC, which was proved to be cytocompatible allowing cells to be readily encapsulated and cultured in these gels [131].

A PEG-based hydrogel with tunable mechanical properties was developed by combination of both photoinitiated thiol-ene chemistry, for the surface functionalization of a PEG-hydrogel, and oxime ligation, for the synthesis of the hydrogel [132].

The radical mediated thiol-ene chemistry was also applied to natural hydrogels. Desai and co-workers developed a click alginate system using photoinitated thiol-ene based modification of norbornene groups to present thiol-bearing peptides. The carboxyl group of alginate was previously modified with norbornene methanamine by carbodiimide chemistry [133].

This type of reaction is an attractive approach for hydrogel formation, in spite of this, thiols, in the presence of oxygen tend to form disulfides, a the major product of thiol oxidation; moreover, the presence of cysteine and amine residues can threaten the process of hydrogel formation [91].

**Figure 5:**
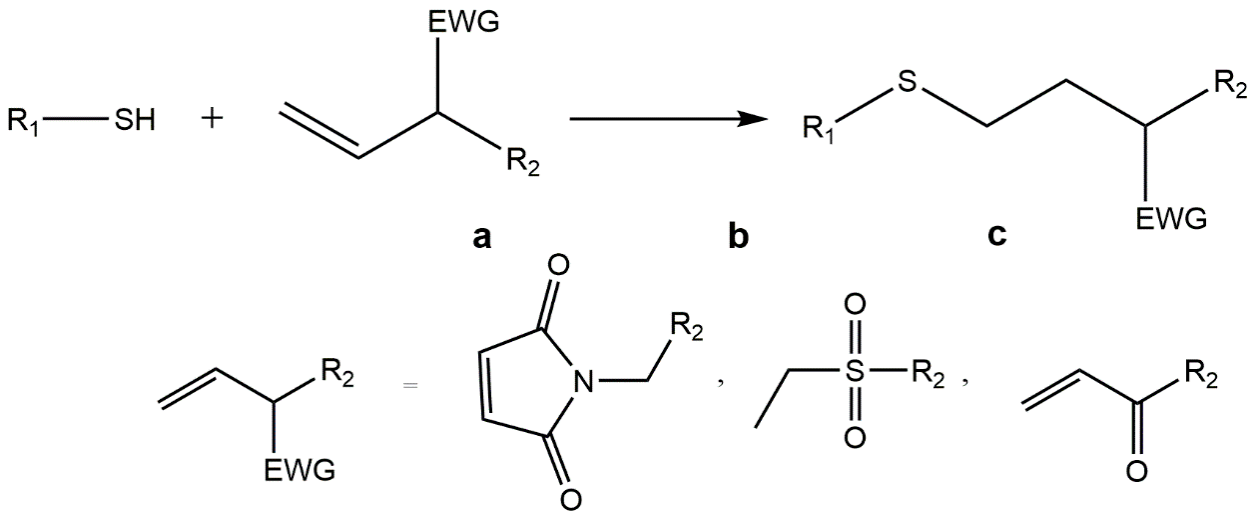
Michael additions can selectively link a thiol group from any peptide (e.g., from a cysteine residue) with an electronically-deficient double bond of, e.g., (**a**) maleimide; (**b**) vinyl sulfone or (**c**) acrylic groups, in a polymer backbone through a stable thioether bond.

Michael addition reactions have been widely used as functionalization tools since they are fast and applicable at low concentrations of reagents. Furthermore, they provide a high selectivity in the presence of common functional groups, ensuring oriented and homogeneous peptide immobilization without affecting materials properties such as stiffness or swelling. Michael additions can selectively link a thiol group from any peptide (e.g., from a cysteine residue) with an electronically deficient double bond of, e.g., maleimide, vinyl sulfone groups or acrylic, in a polymer backbone by creating a stable thioether bond (Figure 5). The nature of the electron-withdrawing group (EWG) on the carbon–carbon double bond influence the overall rate and yield of such reactions. The order of reactivity among types of double bonds in thiol-Michael addition is as follows: maleimide, vinyl sulfone, acrylates/acrylamides, acrylonitrile and methacrylates/methacrylamides [9,134]. Michael additions have been seen as crosslinking strategy to functionalize polymer matrices with proteins, integrin binding peptides and enzymatically degradable linkers under aqueous-buffered conditions [87,91].

Michael addition reactions have been used, for instance, by Tsurkan and colleagues for the functionalization of PEG-heparin hydrogels with various biofunctional peptides preserving the hydrogel network. The reaction proved to be a highly effective and fast strategy to covalently graft peptides onto the surface of hydrogels in a controllable manner [87]. Hubbel and co-workers used thiol-acrylate Michael addition reactions to form drug-delivery hydrogels using; the materials thus produced showed controllable polymerization reactivity and degradability [126,135,136]. The same type of chemoselective reaction was equally used by Anseth and colleagues to develop cell adhesion scaffolds by incorporating thiol-functionalized peptide sequences within the PEG-based hydrogel network, previously modified with methacrylate groups [137].

**Figure 6:**
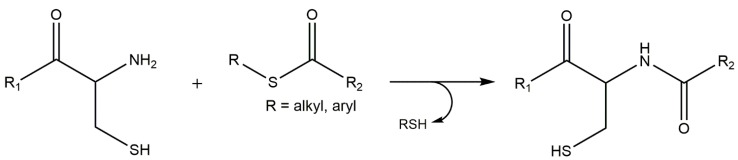
Native chemical ligation (NCL): this reaction proceeds through transesterification of the C-terminal thioester and the N-terminal cysteine to form a new intermediate thioester, in aqueous solution, under mild conditions. This thioester spontaneously rearranges by an S to N acyl shift leading to the desired amid bond.

Michael additions were also used by Su and co-workers to functionalize a cysteine-terminated PEG-based hydrogel with maleimide-terminated peptides. Interestingly, this study used native chemical ligation (NCL, Figure 6), another type of “click” reaction, to previously crosslink the hydrogel. This chemistry proceeds through transesterification of the C-terminal thioester and the N-terminal cysteine to form a new thioester, under mild conditions. This thioester then spontaneously rearranges by an S to N acyl shift, in aqueous environment, leading to the desired amid bond. Current biological applications have been using NCL for cross-linking hydrogel-based scaffolds. Furthermore, this reaction is exceptionally chemo and regioselective, avoiding unwanted side reactions [31,138,139].

### 5.4. Diels–Alder Cycloadditions

The Diels–Alder (DA) [4+2] cycloaddition combines a diene and a substituted alkene, commonly referred to as the dienophile, This is a highly selective reaction that gives a substituted cyclohexene without any catalyst or byproduct, and which is greatly accelerated in water due to increased hydrophobic effects. DA cycloadditions are eventually reversed at high temperature through the retro-DA reaction, which opens a way to controlled drug release [108,140,141].

**Figure 7:**
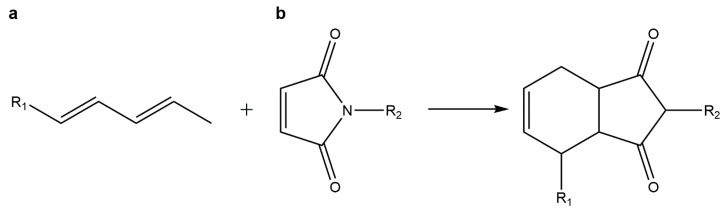
Diels–Alder reaction: in this cycloaddition reaction a (**a**) diene reacts with a (**b**) dienophile yielding a substituted cyclohexene without any catalyst or byproduct.

Amongst DA reactions, the inverse electron demand DA cycloaddition of tetrazine and a dienophile (for example norborene or *trans*-cyclooctene), is known to be a powerful biorthogonal chemistry tool suitable for cell-labelling and occurs. This type of “click” chemistry was also used for covalently cross-link polymer networks, even in the absence of a catalyst or other additives. For example, Alge *et al.* developed a cell-laden hydrogel using a functionalized PEG-based hydrogel with a biologically active ECM-mimetic peptide. Results demonstrated the potential of the tetrazine-norbornene cycloaddition (Figure 7) as an interesting strategy to develop novel hydrogel-scaffolds for cell-culture [142].

### 5.5. Oxime “Click” Chemistry

**Figure 8:**
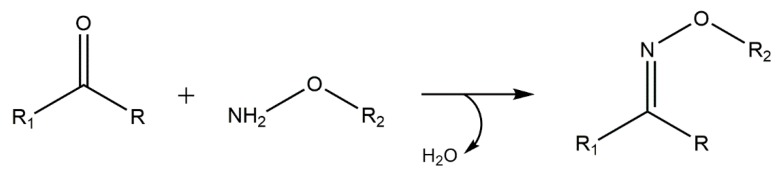
Oxime “click” reaction between an aminooxy group and carbonyl groups.

Oxime “click” reactions occur between an aminooxy group and carbonyl groups of aldehydes or ketones, which are stable when compared to thiols (Figure 8). These are ideal reactions for formation of protein-polymer conjugates, since those reactive groups can be easily incorporated into proteins and peptides. In fact, this biorthogonal reaction has already been used in cell surface modification, and to label biological molecules [91].

The oxime bond formation is fast producing only water as a by-product. Interestingly, the reaction kinetics is pH-sensitive, and also depends on catalyst concentration. These features allows to create hydrogels with tunable properties and varying degrees of reversibility [91,132]. In a recent study by Grover and co-workers, a ketone-modified RGD peptide was used to successfully functionalize an aminooxy PEG hydrogel through oxime chemistry [91].

## 6. Concluding Remarks

Bioactive peptides are highly specific, effective and safe thus representing an interesting alternative to other bioactive drugs. Given the relevance of peptides many methods and strategies have been studied and developed for the chemical synthesis of novel peptides with improved physicochemical properties. Therefore, new classes of peptides, such as cell penetrating peptides and peptide-conjugates, are emerging, broadening the number of possible biomedical applications. The progression of peptide compounds into clinical therapy requires alternatives to their traditional parental administration and also the development of peptide-conjugates, namely to polymer scaffolds [11].

In order to create more effective polymeric peptide carriers, studies are now exploring the potential of combining multiple tethering strategies [132,143]. For example, De Forest *et al.* reported the formation of hydrogels merging two “click” chemistry schemes, from PEG-azides and strained alkyne-flanked peptides followed by a second thiol-ene “click” reaction to incorporate biological functionalities within the gel network [125,129,143]. Polizzotti and colleagues developed a PEG functionalized hydrogel using multiple “click” chemistries, CuAAC for gelation and thiol-ene photocoupling for complex patterning [144]. Consequently, “click” chemistry is showing great promise towards the development of polymer-drug/peptide conjugates of biomedical interest.
